# Signaling Through Girdin Underlies Excessive Cell Morphogenesis Resulting from Depletion of Neurodevelopmental Disorder-Related Neurexin-2

**DOI:** 10.3390/ijms27156612

**Published:** 2026-07-24

**Authors:** Hideji Yako, Mikito Takahashi, Mami Akiyama, Ayaka Suzuki, Yuki Miyamoto, Junji Yamauchi

**Affiliations:** 1 Laboratory of Molecular Neuroscience and Neurology, Tokyo University of Pharmacy and Life Sciences, 1432-1 Horinouchi, Hachioji, Tokyo 192-0392, Japan; hyako@toyaku.ac.jp (H.Y.);; 2 Department of Precision Medicine, National Research Institute for Child Health and Development, Setagaya, Tokyo 157-8535, Japan; 3 Department of Biological Sciences, Tokyo College of Biotechnology, Ota, Tokyo 157-8535, Japan

**Keywords:** NRXN2, NDD, Girdin, ELMO, Rac1, neurite outgrowth

## Abstract

During development, neurexin-2 (NRXN2) is a cell adhesion molecule localized to presynaptic terminals as well as axonal shafts and immature neurites, where it participates in the regulation of neuronal cell morphogenesis. Given its critical role in early neuronal development, NRXN2 is considered a susceptibility gene product for neurodevelopmental disorders (NDDs) such as autism spectrum disorder (ASD) and intellectual disability (ID). However, the intracellular signaling mechanisms linking *NRXN2* deficiency to abnormal neuronal cell morphology remain unclear. Herein, we investigated the molecular basis of excessive cell morphogenesis induced by the knockdown of *NRXN2* using the N1E-115 cell line, a model of neuronal morphogenesis characterized by neurite outgrowth. Silencing *NRXN2* using the clustered regularly interspaced short palindromic repeat (CRISPR)/Cas13 system resulted in a marked enhancement of process elongation. Mechanistically, we found that Girdin (also called GIV or CCDC88A), a non-receptor guanine nucleotide exchange factor for heterotrimeric G proteins, can mediate the excessive process length phenotype. Transfection of either the regulator of G protein signaling (RGS) domain of RGS3, a GTPase-activating protein for G proteins, or the G protein-binding domain of engulfment and cell motility 1 (ELMO1) rescued the excessive process formation. Similar results were obtained in primary cortical neurons. In addition, these interventions normalized downstream Rac1 activity in cells. Together, our findings elucidate Girdin signaling as a mediator of excessive neuronal process formation following NRXN2 knockdown, providing mechanistic insight into how the loss of function of NRXN2 leads to aberrant cell morphogenesis at least at the molecular and cellular levels. These results suggest that signaling through Girdin may contribute to the morphological abnormalities associated with NRXN2-related neurodevelopmental disorders.

## 1. Introduction

The establishment of precise neural circuits in the developing central nervous system (CNS) depends on highly coordinated and dynamic morphological change and/or remodeling of neuronal cells [1,2,3,4]. Throughout development, neuronal cells extend and elongate neurites, navigate toward appropriate targets, and progressively assemble synaptic connections to form organized neuronal networks [5,6]. Despite extensive investigation, the molecular programs that precisely regulate the sequential stages of neuronal cell morphological differentiation remain incompletely defined. Perturbations in these morphogenetic events can arise not only during early neurodevelopment but also at later stages, resulting in aberrant neuronal process extension, guidance, synapse formation, and their maintenance [7,8,9,10].

Such structural abnormalities are thought to compromise circuit assembly by disrupting intercellular connectivity and the neurite elongation required for proper integration within brain regions. In particular, dysregulated control of neuronal process elongation, which may manifest as either excessive [11] or insufficient [12] neurite or axonal growth based on anatomical observations, is thought to play a role in several neurological and neurodevelopmental disorders [11,12,13,14].

Evidence indicates that dysregulation of neuronal cell morphogenesis constitutes a fundamental pathogenic mechanism underlying neurodevelopmental disorders (NDDs), including autism spectrum disorder (ASD) and intellectual disability (ID), ultimately contributing to deficits in neuronal process and network formation, as well as synaptic organization [15,16].

Among the gene products associated with NDDs, members of the neurexin (NRXN) protein family have emerged as important regulators of neuronal development and connectivity [17,18]. All NRXNs are cell adhesion molecules enriched at presynaptic terminals that interact transsynaptically with neuroligins and other postsynaptic protein partners to organize synaptic structure and regulate neurotransmission [17,18]. While NRXNs are widely recognized for their roles in synaptic adhesion and transmission, accumulating evidence shows that they also participate in earlier stages of neuronal morphogenesis preceding synaptogenesis [17,18,19,20]. Indeed, during development, NRXNs, including NRXN2, are localized not only to presynaptic terminals but also to axonal shafts and immature neurites, where they function as cell adhesion molecules involved in the regulation of neuronal cell morphogenesis [19,20]. *NRXN2* has been identified as a susceptibility gene for ASD and ID primarily through loss-of-function mutations [21,22]; however, the intracellular signaling pathways linking *NRXN2* deficiency to abnormal neuronal cell morphology remain to be defined.

It is known that heterotrimeric G proteins and their modulators coordinate diverse signaling pathways that influence cell morphogenesis through intermediate signaling molecules such as the Rho family small GTPase Rac1 [23,24]. Girdin (also known as GIV or CCDC88A) is a non-receptor guanine nucleotide exchange factor for heterotrimeric G proteins [25,26]. Girdin has been implicated in cell and tissue development [25,26]. Therefore, Girdin has been shown to interact, both physically and cell biologically, with signaling cascades related to Rac1, a master regulator of cell morphological changes [27,28].

Given the importance of balanced signaling for neuronal process formation, dysregulation of G protein and Rac1 signaling pathways may represent a mechanistic link between NRXN2 loss of function and aberrant process formation. We therefore hypothesized that *NRXN2* knockdown might disrupt intracellular signaling networks, leading to abnormal process elongation through a Girdin-dependent mechanism.

In the present study, we examined the molecular basis of enhanced neuronal morphogenesis induced by *NRXN2* knockdown using the mouse N1E-115 neuronal cell line [29,30] and mouse primary cortical neurons [31,32]. By combining clustered regularly interspaced short palindromic repeat (CRISPR)/Cas13-mediated knockdown [33,34] with transfection of G protein signaling components (the regulator of G protein signaling [RGS] domain of RGS3 or the G protein-binding domain of engulfment and cell motility 1 [ELMO1]) [35,36,37,38,39,40], we identify Girdin as a key mediator of excessive process elongation following *NRXN2* knockdown. Furthermore, we demonstrate that modulation of heterotrimeric G protein regulators restores downstream Rac1 activity to normal levels. Our findings provide mechanistic insight into how impaired NRXN2 signaling may lead to aberrant cell morphology and suggest that Girdin signaling can contribute to morphological abnormalities in NRXN2-related NDDs.

## 2. Results

### 2.1. Knockdown of NRXN2 in Cells Leads to Activation of Girdin

Girdin is a multifunctional non-receptor guanine nucleotide exchange factor for G proteins that acts as a signaling hub beneath the plasma membrane, coordinating upstream signaling pathways controlling cell morphogenesis [25,26].

**Figure 1:**
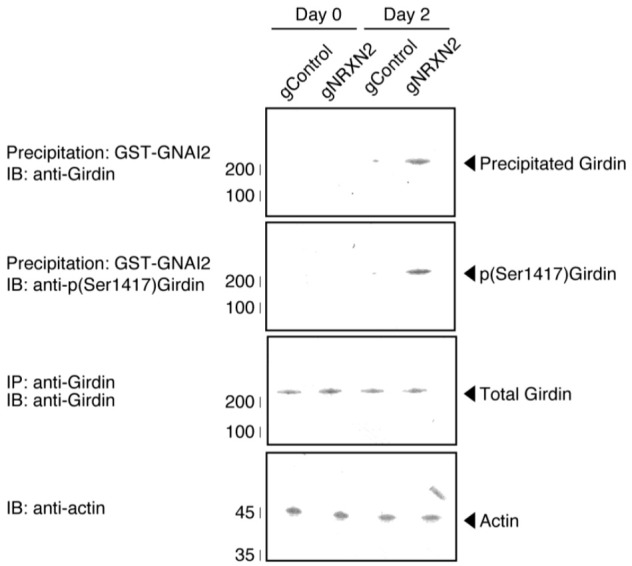
Affinity-precipitation of Girdin following depletion of NRXN2. N1E-115 cells were transfected with gRNA to Girdin targeting NRXN2 or control gRNA and Cas13. The lysates were used for an affinity-precipitation assay with guanine-nucleotide-free GNAI2. They were subjected to immunoblotting using antibodies against p(Ser1417) Girdin, Girdin, or actin after immunoprecipitation performed either with or without an anti-Girdin antibody.

We therefore sought to determine how the activity of Girdin is affected by *NRXN2* knockdown. To address this, we first knocked down *NRXN2* in N1E-115 cells. Transfection of plasmids encoding CRISPR/Cas13-fitted gRNA specific for *NRXN2*, but not control gRNA, together with Cas13, resulted in decreased NRXN2 protein expression (Figure S1). Using this system, we examined the effect of *NRXN2* knockdown on Girdin. We performed an affinity-precipitation assay for Girdin protein under NRXN2 or control gRNA transfection conditions. Since the activated form of guanine nucleotide exchange factors binds to a guanine nucleotide-free form of the effector protein [25,26,40], we performed affinity precipitation of active Girdin using recombinant GST-tagged, guanine nucleotide-free GNAI2 protein. As a result, knockdown of *NRXN2*, but not its control, increased the amount of affinity-precipitated Girdin (Figure 1). Also, the precipitated Girdin protein was phosphorylated at Ser-1417, a site critical for its activation [25,26], indicating that reduced expression of NRXN2 leads to activation of Girdin.

**Figure 2:**
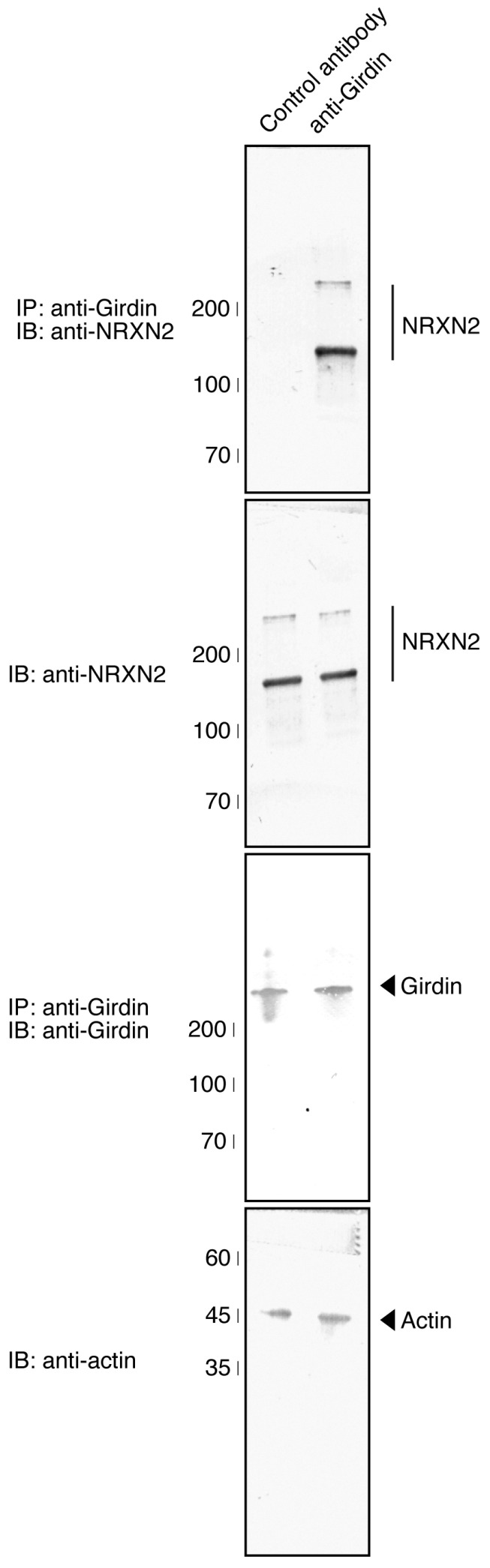
Interaction of NRXN2 with Girdin in cells. N1E-115 cell lysates were subjected to immunoprecipitation with an anti-Girdin antibody, followed by immunoblot analysis using either an anti-NRXN2 antibody or an anti-Girdin antibody to detect total Girdin. The lysates was also immunoblotted using an antibody against NRXN2 or actin.

Furthermore, to determine whether NRXN2 interacts with Girdin, we performed a co-immunoprecipitation assay. NRXN2 was detected in Girdin immunoprecipitates, indicating that these two proteins form a complex in cells (Figure 2).

### 2.2. Girdin Signaling Mediates the Excessive Process Elongation Phenotype

Since knockdown of *NRXN2* specifically resulted in upregulation of Girdin, we hypothesized that this increase may be associated with the promotion of excessive cell morphological changes. To test this, we used *NRXN2*-knockdown cells and allowed their cells to differentiate. As expected, the knockdown significantly promoted excessive process elongation, consistent with increased expression of neuronal differentiation markers, growth-associated protein 43 (GAP43) and β3 tubulin (Figure S2). In contrast, following differentiation, the expression levels of internal control protein were comparable between knockdown conditions.

**Figure 3:**
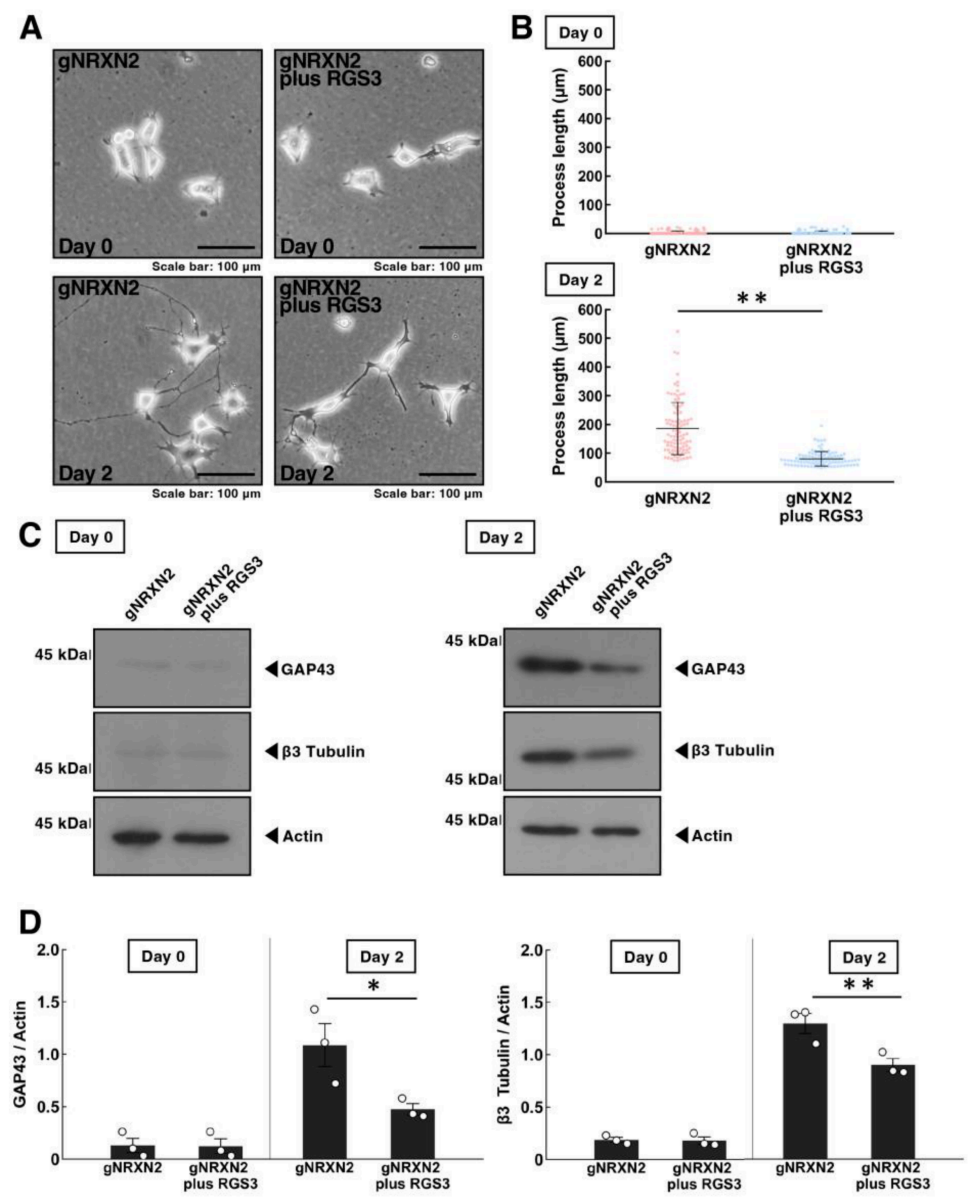
RGS3 recovers the excessive process elongation phenotype. (**A**,**B**) Plasmids encoding the RGS domain of RGS3 (hereafter referred to as RGS3) or the corresponding control vector were transfected into N1E-115 cells stably harboring gRNA targeting NRXN2 and Cas13. After induction of differentiation, cells were cultured for 0 or 2 days. Representative images are shown. The length of the longest process was measured and depicted statistically (** *p* < 0.01; *n* = 100 cells). (**C**,**D**) Lysates from cells at day 0 or day 2 following the induction of differentiation were immunoblotted with antibodies against neuronal differentiation marker proteins GAP43 and b3 tubulin or the internal control marker protein actin. The band intensity of each marker protein was normalized to that of actin and statistically analyzed (** *p* < 0.01 and * *p* < 0.05; *n* = 3 blots). Vertical lines in the graph indicate the groups being compared.

To investigate whether G proteins acting as Girdin effectors contribute to the formation of this phenotype, we transfected the plasmid encoding the RGS domain of RGS3, a GTPase-activating protein for many types of G proteins [35], into cells. Notably, expression of the RGS domain of RGS3 effectively suppressed the excessive process elongation phenotype induced by *NRXN2* knockdown, restoring it toward control levels (Figure 3A,B). These results were supported with decreased levels of neuronal protein markers GAP43 and β3 tubulin (Figure 3C,D). In contrast, under control knockdown conditions, cell morphology and the expression levels of neuronal protein markers were comparable between cells transfected with the RGS3 construct and those transfected with the control vector (Figure S3).

**Figure 4:**
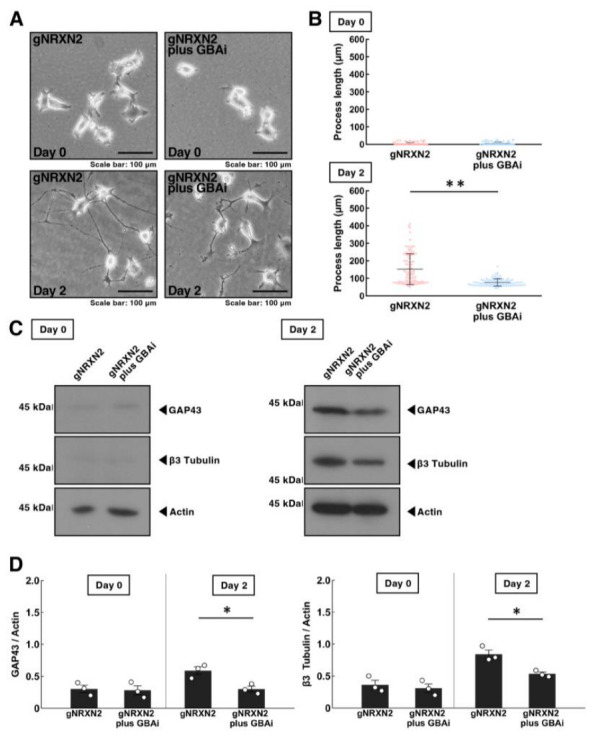
GBAi, a Girdin inhibitor, recovers the excessive process elongation phenotype. (**A**,**B**) Plasmids encoding GBAi or the corresponding control vector were transfected into N1E-115 cells harboring gRNA targeting NRXN2 and Cas13. After induction of differentiation, cells were cultured for 0 or 2 days. Representative images are shown. The length of the longest process was measured and depicted statistically (** *p* < 0.01; *n* = 100 cells). (**C**,**D**) Lysates from cells at day 0 or day 2 post-differentiation were immunoblotted with antibodies against neuronal differentiation marker proteins GAP43 and b3 tubulin or an internal control marker protein actin. The band intensity of each marker protein was normalized to that of actin and statistically analyzed (* *p* < 0.05; *n* = 3 blots). Vertical lines in the graph indicate the groups being compared.

We next examined whether direct inhibition of Girdin signaling could attenuate the excessive morphological phenotype. We utilized GBAi, an inhibitor of the interaction between Girdin and G protein GNAI subfamily proteins [36]. Transfection of a plasmid encoding GBAi suppressed the excessive process elongation induced by *NRXN2* knockdown (Figure 4A,B). Furthermore, the expression levels of neuronal differentiation markers, including GAP43 and β3 tubulin, were concomitantly decreased following transfection of GBAi (Figure 4C,D), supporting the notion that Girdin inhibition dampens the aberrant differentiation program triggered by *NRXN2* knockdown. In contrast, under control knockdown conditions, cell morphology and the expression levels of neuronal protein markers were comparable between cells transfected with the GBAi construct and those transfected with the control vector (Figure S4).

Taken together, these results support the idea that Girdin acts downstream of NRXN2 knockdown phenotypes. Similar morphological changes observed in N1E-115 cells were also observed in primary cortical neurons (Figure S5).

### 2.3. ELMO1’s G Protein-Binding Domain Restores the Excessive Process Elongation Phenotype

Girdin is known to positively regulate Rac1 to promote cell morphogenesis in cell types other than neuronal cells [37,38]. Additionally, activation of Rac1 through the ELMO-DOCK signaling complex preferentially plays a role in neurite elongation in N1E-115 cells and primary cortical neurons [39]. Therefore, we explored whether ELMO1’s G protein-binding domain (amino acids 81–310), which is responsible for coupling G proteins to signals mediated by DOCK-A and -B subfamily molecules [40], can reduce the excessive process elongation induced by *NRXN2* knockdown.

**Figure 5:**
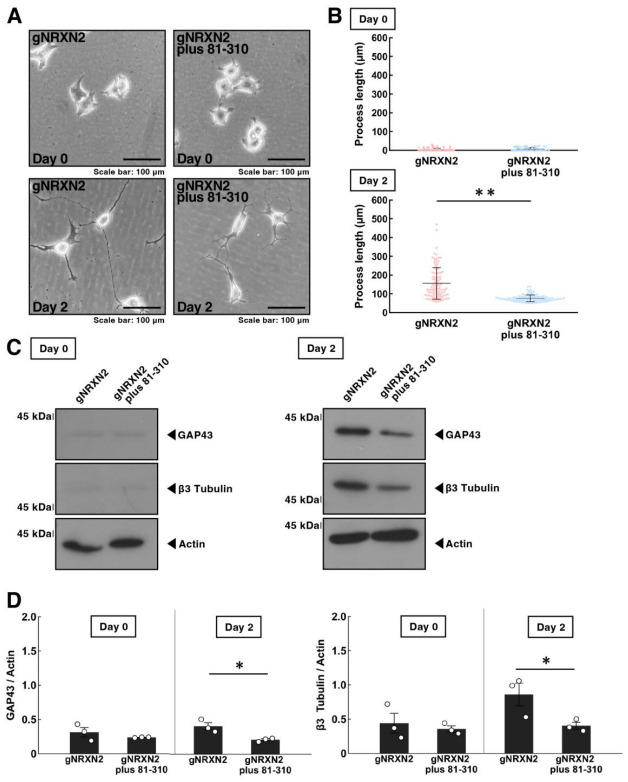
Amino acids 81–310 of ELMO1 (G protein-binding domain) recovers the excessive process elongation phenotype. (**A**,**B**) Plasmids encoding amino acids 81–310 of ELMO1 (hereafter referred to as 81–310) or the corresponding control vector were transfected into N1E-115 cells harboring gRNA targeting NRXN2 and Cas13. After induction of differentiation, cells were cultured for 0 or 2 days. Representative images are shown. The length of the longest process was measured and depicted statistically (** *p* < 0.01; *n* = 100 cells). (**C**,**D**) Lysates from cells at day 0 or day 2 post-differentiation were immunoblotted with antibodies against neuronal differentiation marker proteins GAP43 and b3 tubulin or an internal control marker protein actin. The band intensity of each marker protein was normalized to that of actin and statistically analyzed (* *p* < 0.05; *n* = 3 blots). Vertical lines in the graph indicate the groups being compared.

Transfection of the G protein-binding domain into N1E-115 cells suppressed both excessive elongation (Figure 5A,B) and the increased expression of neuronal marker proteins (Figure 5C,D). Similar results were observed in primary cortical neurons (Figure S5). In contrast, under control knockdown conditions, cell morphology and the expression levels of neuronal protein markers were comparable between cells transfected with the G protein-binding domain construct and those transfected with the control vector (Figure S6).

### 2.4. Elevated Rac1 Activity Was Restored to Normal Levels by Transfection of Each Inhibitory Molecule Used in the Preceding Experiments

**Figure 6:**
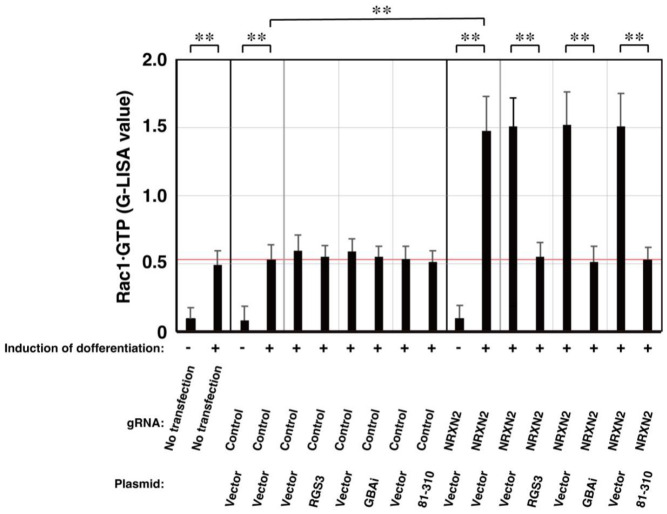
Each G protein construct recovers excessive Rac1 activation. N1E-115 cells stably harboring gRNA targeting NRXN2 or control gRNA and Cas13 were transfected with plasmids encoding the respective constructs or its empty vector (vector) and cultured for 2 days with (+) or without (−) differentiation induction. Cell lysates were analyzed using a G-LISA GTPase kit to assay the amounts of GTP-bound Rac1 (** *p* < 0.01; *n* = 10). Vertical lines in the graph indicate the groups being compared. In addition, a red reference line indicating the Rac1 activity level of control differentiated cells was added.

We further examined whether each inhibitory molecule used in the preceding experiments could attenuate the activity of Rac1, a master regulator of cell morphogenesis, to normal levels in N1E-115 cells. We transfected plasmids encoding the RGS domain, GBAi, or the G protein-binding domain into control- or NRXN2-knockdown cells and assayed the activity of Rac1 (Rac1·GTP levels). All constructs restored Rac1 activity in *NRXN2*-knockdown cells to levels comparable to those in control cells (Figure 6). These results indicate that the inhibitory molecules used in the preceding experiments attenuate the elevated Rac1 activity induced by *NRXN2* knockdown, supporting the involvement of signaling mediated by the Girdin-G protein-ELMO axis in this molecular mechanism.

## 3. Discussion

Aberrant regulation of neuronal process elongation, characterized by either excessive or impaired growth, is considered a contributing factor in a range of neurological and neurodevelopmental disorders [41,42]. Accumulating evidence suggests that abnormalities in neuronal morphogenesis represent a core pathogenic mechanism in NDDs such as ASD and ID [41,42,43,44]. In this study, we explored the molecular mechanisms underlying abnormal neuronal morphogenesis caused by *NRXN2* knockdown and identified Girdin as a critical mediator of excessive process elongation under our experimental conditions.

NRXN2 is a single-pass transmembrane adhesion molecule that is well characterized as a presynaptic cell adhesion molecule involved in synapse formation, maintenance, and function [21,22]. Additionally, during development, and possibly during limited neuritegenesis in adulthood, NRXN2 localizes to axonal shafts and immature neurites, where it acts as a cell adhesion molecule that regulates neuronal cell morphogenesis [19,20]. Our findings confirm that NRXN2 plays a key role in neuronal cell morphological differentiation and demonstrate that its knockdown leads to excessive cell morphogenesis.

One of the central findings of this study is that Girdin signaling mediates the enhanced process elongation induced by NRXN2 knockdown. Girdin acts as a non-receptor guanine-nucleotide exchange factor or heterotrimeric G proteins and serves as a key integrator of diverse signaling pathways regulating cell morphogenesis, migration, and polarity [25,26]. Its involvement for the observed phenotype suggests that *NRXN2* knockdown disrupts intracellular signaling homeostasis, leading to aberrant activation of signaling pathways dependent on G proteins. This finding may highlight a conceptual link between the precise regulation of cell adhesion molecules and intracellular signaling networks.

**Figure 7:**
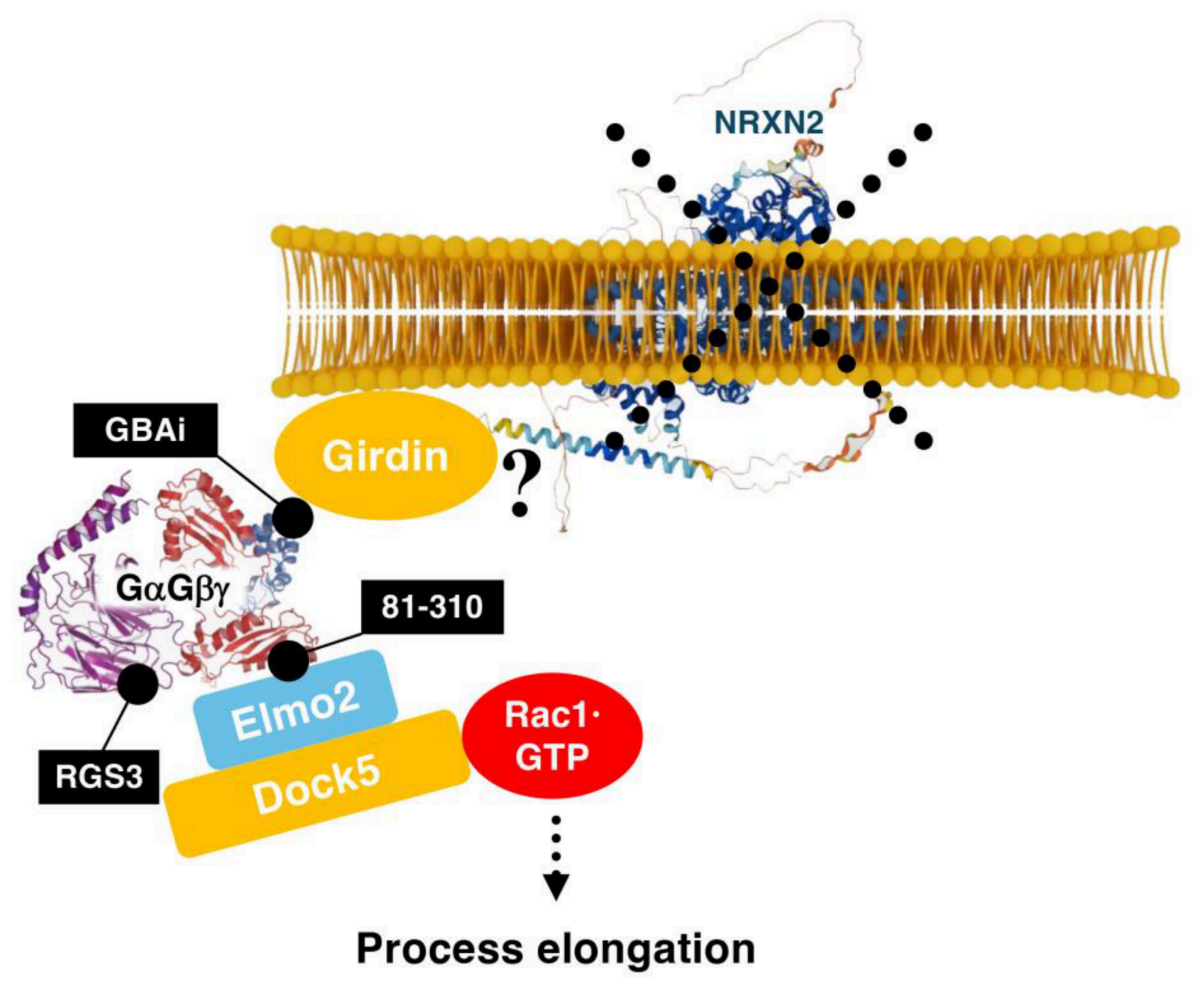
Proposed signaling model underlying the neuronal morphological changes induced by NRXN2 knockdown. NRXN2 knockdown leads to the activation of Girdin, which can subsequently link to heterotrimeric G proteins (A dashed × indicates NRXN2 knockdown). In our previous study [39], one of the downstream effectors, ELMO2, was shown to associate with DOCK5 to activate Rac1, thereby contributing to process elongation under experimental pathological conditions. Although NRXN2 forms an immunoprecipitated complex with Girdin, the functional interaction between NRXN2 and Girdin remains unclear. GBAi, an inhibitor of Girdin; RGS3, an accelerator domain for the inactivation of heterotrimeric G proteins; and 81–310, a region of ELMO2 serving as the binding site for heterotrimeric G proteins. The 3D models of mouse NRXN2 and mouse heterotrimeric G protein were generated using AlphaFold2 (based on PBD Nos. pdb_00003bop and pdb_00006crk). They are described as conceptual representations for illustrative purposes only.

ELMO and DOCK proteins form a bipartite guanine nucleotide exchange factor complex that activates Rac1, thereby controlling cell morphogenesis [40]. The ELMO2 and DOCK5 protein complex is involved in the fine-tuning of neuronal process elongation [39]. ELMO2 protein interacts with diverse upstream signaling molecules, including small GTPases and G protein subunits, and contributes to DOCK5 protein activation by integrating these signals [39]. The present study reveals a complex relationship between G protein signaling and Rac1 activity. Transfection of the RGS domain of RGS3, a negative regulator of heterotrimeric G proteins, or the G protein-binding domain restored the excessive process elongation phenotype. These manipulations also normalized Rac1 activity (see the schematic diagram of Figure 7). While the association between morphological outcomes and Rac1 activity suggests that Rac1 is an important determinant of process elongation, it is likely that Girdin and G proteins specifically contribute to excessive elongation under pathological conditions.

From a disease perspective, the identification of Girdin-mediated excessive process elongation following *NRXN2* knockdown may provide a previously unrecognized framework for understanding the structural basis of NDD pathogenesis. While previous research shows largely characterized NRXNs, including NRXN2, as mediators of synaptic maintenance [45,46,47,48], our data can emphasize their earlier and crucial role as morphogenetic brakes during initial process elongation.

In the context of ASD and ID, brain hyperconnectivity and macrocephaly are frequently observed clinical features [45,46,47,48]. At the cellular level, these may manifest not only as an increase in synapse number but also as aberrant axonal and/or dendritic elongation [45,46,47,48]. Our results suggest that the excessive morphogenesis observed upon *NRXN2* knockdown, driven by dysregulation of the Girdin-G protein-Rac1 axis, could lead to the formation of ectopic neuronal networks. If neuronal processes elongate beyond their physiological targets due to a failure of termination signals, they may establish improper transsynaptic partnerships, increasing the structural noise within local cortical micronetworks. Such miswiring is indeed a recognized contributor to the imbalance between excitatory and inhibitory signaling, a hallmark of ASD and ID pathophysiology [45,46,47,48].

The mechanistic involvement of Girdin is particularly salient. As a non-receptor guanine nucleotide exchange factor for G proteins, Girdin serves as a versatile signaling hub that bridges cell surface adhesion molecules with cell morphogenesis regulated by Rac1 [25,26,27,28]. In our model, the knockdown of *NRXN2* appears to release an inhibitory constraint on Girdin, possibly leading to activation of G protein subunits. This is consistent with our observation that NRXN2 and Girdin form a protein complex in cells, suggesting that NRXN2 may directly sequester or inhibit Girdin. In turn, this can recruit the ELMO-DOCK complex to possible, specific intracellular regions, triggering the overactivation of Rac1. The ability of the RGS domain of RGS3 or the G protein-binding domain to rescue the cellular phenotype supports the idea that this specific signaling bottleneck may be responsible for the pathological overgrowth. Furthermore, the consistency of these findings across both the N1E-115 cell line and primary cortical neurons could underscore the robustness of this signaling axis.

Looking forward, this study positions the NRXN2-Girdin-G protein pathway as a potential therapeutic target of NRXN2-related diseases at least at the molecular and cellular levels. Small molecules or peptides designed to disrupt the Girdin-G protein-ELMO interaction, or those that enhance RGS protein activity, might serve as molecular stabilizers to normalize neuronal process elongation in individuals carrying loss-of function *NRXN2* mutations. Future studies using CRISPR/Cas13 or Cas9-mediated *NRXN2* depletion in embryonic mouse brains would be essential to determine whether these morphological abnormalities directly translate into the behavioral and cognitive deficits characteristic of ASD and ID. The possible downstream signaling modification could recover these phenomena. By shifting the focus from synaptic terminals to early stages of neuronal process navigation, this work can help identify earlier windows for intervention in NDDs related to *NRXN2*.

## 4. Materials and Methods

### 4.1. Key Materials

Key materials used in this study are listed in Table 1.

### 4.2. Cell Line Culture and Differentiation

The mouse neuronal N1E-115 cell line was maintained in culture on cell culture dishes (Thermo Fisher Scientific, Waltham, MA, USA) in high-glucose Dulbecco’s modified Eagle medium (DMEM, Nacalai Tesque, Kyoto, Japan) supplemented with 10% heat-inactivated fetal bovine serum (FBS, Thermo Fisher Scientific) and penicillin–streptomycin, under a humidified atmosphere of 5% CO_2_ at 37 °C, as previously described [39]. Stable cells expressing *NRXN2* gRNA cloned into pSINsi-mU6 (Takara Bio, Kyoto, Japan) together with a Cas13 expression plasmid (Addgene, Watertown, MA, USA) were generated by selection with G418 (0.2 mg/mL) for 2 weeks, following the manufacturer’s instructions [39]. The established clones were continuously maintained in culture without cryopreservation.

Cells were induced to differentiate by culture in DMEM supplemented with 1% FBS and penicillin–streptomycin at 37 °C in a humidified atmosphere containing 5% CO_2_ for 0 or 2 days, unless otherwise specified. Following induction, the length of the longest cellular processes was measured in a blinded manner using ImageJ (version Java 8; NIH, Bethesda, MD, USA) with the NeuronJ add-in (version 1.4.3, NIH). Data are presented as dot plots generated using R (version 4.6.0; R Foundation for Statistical Computing, Vienna, Austria).

Under these conditions, fewer than 5% of adherent cells were positive for trypan blue staining in each experiment.

### 4.3. Primary Cell Culture and Axon Elongation

Animals were handled in accordance with the ARRIVE guidelines (https://arriveguidelines.org/ accessed on 4 January 2025), and all studies were conducted according to committee-approved protocols as described in the Ethics statement section. Mice were euthanized by chemical anesthesia using an intraperitoneal injection of a mixture of 0.3 mg/kg medetomidine, 4 mg/kg midazolam, and 5 mg/kg butorphanol.

Primary cortical neurons were prepared from the cerebral cortex of C57BL/6JJcl mouse embryos at embryonic days 16–17, as described previously. Briefly, tissues were incubated with papain (100 U/mL) in Leibovitz’s L-15 medium at 37 °C for 15 min, followed by gentle trituration to obtain a single-cell suspension [31,32]. The dissociated cells were subsequently seeded onto poly-L-lysine–coated culture dishes at a density of 3–5 × 10^5^ cells/cm^2^.

Neurons were cultured in Neurobasal medium supplemented with 2% B27 (Thermo Fisher Scientific), 1% GlutaMAX (Thermo Fisher Scientific), and 0.1 mg/mL gentamicin. Cells were maintained at 37 °C in a humidified atmosphere containing 5% CO_2_. The culture medium was completely replaced 24 h after plating, and thereafter, half of the medium was refreshed every 2–3 days. After 7–14 days in vitro, cortical neurons were dissociated using 0.05% trypsin containing 0.53 mM EDTA and preserved in liquid nitrogen until further use.

For process elongation assays, cells were replated onto culture dishes and cultured for several days to permit the extension of cellular processes. Following induction of differentiation, the length of the longest process (axon) was measured in a blinded manner using ImageJ software with the NeuronJ add-in. Data are presented as dot plots.

Under these conditions, fewer than 5% of attached cells were trypan blue-positive in each experiment.

### 4.4. Plasmid Transfection

Cells were transfected with the indicated mammalian expression plasmids based on pcDNA3.1 (+) or pCMV5. They were also employed to generate a stable cell line or transiently expressing cells by co-transfecting a mammalian expression vector encoding Cas13 along with the corresponding gRNAs inserted into a Pol III–driven transcription vector [30,39]. Transfections were performed using the ScreenFect A transfection kit (Fujifilm Wako Chemicals, Tokyo, Japan). They were carried out in serum- and antibiotic-free DMEM according to the manufacturer’s protocol. Serum was typically supplemented 4 h after transfection, and the culture medium was replaced 24 h post-transfection. Cells were subsequently allowed to differentiate for 0 or 48 h, as required for cell biological or biochemical analyses, unless otherwise specified.

Under these conditions, fewer than 5% of attached cells were estimated to be trypan blue-positive in each experiment.

### 4.5. Polyacrylamide Gel Electrophoresis and Immunoblotting

Cells were lysed in lysis buffer (50 mM HEPES-NaOH, pH 7.5, 150 mM NaCl, 5 mM MgCl_2_, 1 mM dithiothreitol [DTT], 1 mM phenylmethane sulfonylfluoride [PMSF], 0.02 mM leupeptin, 1 mM EDTA, 1 mM Na_3_VO_4_, 10 mM NaF, and 0.5% NP-40) [39]. Under standard denaturing conditions, the collected supernatants were mixed with Laemmli sample buffer (Nacalai Tesque) and denatured. Proteins were separated using sodium dodecyl sulfate-polyacrylamide gel electrophoresis (SDS-PAGE) on precast gel (Nacalai Tesque). Following electrophoresis, proteins were transferred onto a polyvinylidene fluoride (PVDF) membrane (Merck, Darmstadt, Germany), blocked with Blocking One (Nacalai Tesque), and subjected to immunoblotting with primary antibodies and horseradish peroxidase (HRP)-conjugated secondary antibodies. Immunoreactive signals were visualized using X-ray film (Fuji Film, Tokyo, Japan) or TMB substrate (Nacalai Tesque). They were recorded with a CanoScan LiDE4000 scanner (Canon, Tokyo, Japan) and CanoScan software (version 2025; Canon). Representative blots from three independent experiments (see Figures S7–S16) are shown. In some cases, band intensities were quantified relative to control signals using ImageJ software.

### 4.6. Affinity-Precipitation Assay for Active Girdin

Cells were lysed in extraction buffer (50 mM HEPES-NaOH, pH 7.5, 150 mM NaCl, 1 mM MgCl_2_, 1 mM DTT, 1 mM PMSF, 0.02 mM leupeptin, 10 mM EDTA, 1 mM Na_3_VO_4_, 10 mM NaF, and 0.5% NP-40) [30,39]. Centrifuged cell supernatants were incubated for 15 min with Glutathione-Sepharose 4B (Chicago, IL, USA) preloaded with GST-tagged guanine nucleotide–free G protein subunit αi2 (GNAI2, Proteintech, Rosemont, IL, USA). The Sepharose -protein complexes were recovered by low-speed centrifugation and washed once with lysis buffer. After resuspension in sample buffer, the samples were subjected to SDS-PAGE followed by immunoblotting to detect precipitated active Girdin along with total Girdin.

### 4.7. Assay for Active GTP-Bound Rac1

Cells were lysed in G-LISA cell lysis buffer (G-LISA GTPase kit, Cytoskeleton, Inc., Denver, CO, USA), according to the manufacturer’s protocol. Briefly, centrifugally collected cell supernatants (same amounts of samples) were incubated in G-LISA affinity capture wells. After washing, wells were incubated with Rac1-specific antibodies. Washed wells were incubated with HRP enzyme-conjugated secondary antibodies and colorimetrically detected using peroxidase reaction reagents.

### 4.8. Statistical Analyses

Values are expressed as the mean ± standard deviation (SD) from independent experiments. Comparisons between two groups were conducted using an unpaired Student’s *t*-test implemented in Microsoft Excel (ver. 2023; Microsoft, Redmond, WA, USA). For multiple group comparisons, one-way analysis of variance (ANOVA) followed by Tukey’s honestly significant difference (HSD) post hoc test was performed using the StatPlus Excel add-in (ver. 2021; AnalystSoft, Alexandria, VA, USA). A *p*-value of fewer than 0.05 was considered statistically significant. A relatively small sample size (*n* = 3 biological replicates for some experiments) represents a limitation of the present study, as it may limit the evaluation of data normality and statistical power. Therefore, the statistical results should be interpreted with caution.

### 4.9. Ethics Statement

All animal experiments were conducted in accordance with protocols approved by the Tokyo University of Pharmacy and Life Sciences Animal Care and Use Committee (No. L24-20, approved 1 April 2026). Gene experiments were conducted according to protocols approved by the Tokyo University of Pharmacy and Life Sciences Gene Committee (No. LSR3-01, approved 1 April 2026).

## 5. Conclusions

Collectively, this study identifies Girdin signaling as a key mediator of excessive neuronal process elongation induced by *NRXN2* knockdown. Our findings emphasize the importance of tightly regulated signaling networks in controlling neuronal cell morphology and suggest that disruption of these networks may contribute to the pathogenesis of NRXN2-related NDDs. Future studies aimed at elucidating the in vivo relevance of the NRXN2-Girdin-G protein, and its interaction with other signaling pathways, would further advance our understanding of molecular and cellular dysregulation in NRXN2-related NDDs.

## 6. Limitations of This Study

This study has several limitations that should be considered when interpreting the findings.

First, our conclusions are based on in vitro systems, including N1E-115 cells and primary cortical neurons. Although these models are useful for dissecting molecular mechanisms, they do not fully reflect the complexity of in vivo neuronal tissue formation. In particular, it remains unclear whether the excessive neuronal process elongation observed following *NRXN2* knockdown directly leads to abnormal neuronal connectivity or behavioral phenotypes. Validation using *NRXN2*-related disease model animals will therefore be necessary to determine the in vivo relevance of the proposed mechanism.

Second, the present study does not include human-derived cellular models. Given the heterogeneity and species-specific aspects of NDDs, it is important to examine whether similar phenotypes are observed in human systems. Future studies using induced pluripotent stem cell (iPSC)-derived neurons would help establish the translational relevance of our findings.

Third, although we have identified Girdin as a key mediator of excessive process elongation, the detailed molecular mechanism linking NRXN2 to Girdin remains unresolved. Specifically, the mechanism by which NRXN2 regulates or constrains Girdin activity has not been determined. Identification of the intermediate signaling components and clarification of the regulatory mechanism between NRXN2 and Girdin is important for a more complete understanding of this pathway. Accordingly, elucidating the molecular mechanism linking NRXN2 to Girdin can be a major focus of our future studies.

Fourth, some quantitative analyses were performed using a limited number of biological replicates (*n* = 3). Although the observed effects were consistently reproduced across independent experiments, the small sample size may limit the assessment of data distribution and statistical power. Future studies with larger sample sizes will be necessary to further validate these findings.

## Figures and Tables

**Table 1:** Key materials used in this study.

Reagents or Materials	Companies or Sources	Cat. Nos.	Concentrations Used
Key antibodies			
Ant-growth-associated protein 43 (GAP43)	Santa Cruz Biotechnology (Santa Cruz, CA, USA)	sc-17,790	Immunoblotting (IB), 1:5000
Anti-beta 3 type tubulin	Santa Cruz Biotechnology	sc-51,670	IB, 1:500
Anti-actin (also called pan-beta type actin)	MBL (Tokyo, Japan)	M177-3	IB, 1:5000
Anti-Girdin	Thermo Fisher Scientific (Waltham, MA, USA)	MA5-47,719	IB, 1:250, immunoprecipitation (IP), 1 microgram for 1 mg of total proteins
Anti-phosphorylated Ser-1416 of Girdin (p[Ser1416]Girdin)	Thermo Fisher Scientific	PA5-143,702	IB, 1:250
Anti-neurexin 2 (NRXN2)	Proteintech (Rosemont, IL, USA)	30,490-1-AP	IB, 1:250
Anti-rabbit IgG (goat) pre-absorbed peroxidase-conjugate secondary antibody	Nacalai Tesque (Koyo, Japan)	21,858-24	IB, 1:10,000
Anti-mouse IgG (goat) pre-absorbed peroxidase-conjugate secondary antibody	Nacalai Tesque	21,860-61	IB, 1:10,000
Recombinant DNAs			
pCMV-SV40ori	A control construct was generated using Addgene Cat. No. 72,035 as a template	N/A	1.25 mg of DNA per 6 cm dish
NLS-RfxCasRx-NLS (mamalian cell expression plasmid for Cas13)	Addgene (Watertown, MA, USA)	109,049	1.25 micrograms of DNA per 6 cm dish
pSINsi-mU6	A control construct was generated using GenScript Cat. No. J4052QWTG0 as a template	N/A	1.25 micrograms of DNA per 6 cm dish
pSINsi-mU6-gRNA for NRXN2 (targeting nucleotide 5064), which has no significant homology to other mouse transcripts	GenScript (Piscataway, NJ, USA)	J4052QWTG0	1.25 micrograms of DNA per 6 cm dish
pcDNA3.1(+)-N-eGFP-human RGS3-RGS domain (amino acids 776-917)/GTPase-activating protein (GAP) domain	GenScript	J761U166G0	1.25 micrograms of DNA per 6 cm dish
pLic-myc-GBAi, which acts as inhibitor of Girdin signaling	Addgene	171,753	1.25 micrograms of DNA per 6 cm dish
pcDNA3.1(+)-N-eGFP-human Elmo1 (amino acids 81–310), which interacts with heterotrimeric G protein subunits	GenScript	J336RUADG0-2	1.25 micrograms of DNA per 6 cm dish
pEGFP-C1 (mamalian cell GFP expression plasmid)	Takara Bio (Kyoto, Japan; discontinued product)	N/A	1.25 micrograms of DNA per 6 cm dish

## Data Availability

The datasets used and/or analyzed for the current study are available from the corresponding author upon reasonable request.

## Supplementary Materials

The following supporting information can be downloaded at https://www.mdpi.com/article/10.3390/ijms27156612/s1.
