# Supplementary material for: Signaling Through Girdin Underlies Excessive Cell Morphogenesis Resulting from Depletion of Neurodevelopmental Disorder-Related Neurexin-2
Source: Int J Mol Sci. 2026 Jul 24;27(15):6612. doi: 10.3390/ijms27156612 (PMC13465888; doi:10.3390/ijms27156612)
Supplement: Supplementary file 1 [file ijms-27-06612-s001.zip › Supplemental figure legends.pdf]

Supplemental figure legends

Figure S1. Knockdown of NRXN2 in cells. N1E-115 cells were transfected with gRNA targeting NRXN2 or control gRNA and Cas13. The lysates immunoblotted using antibodies against NRXN2 or actin.

Figure S2. The effects of control or *NRXN2* knockdown on N1E-115 cells. (A, B) N1E-115 cells stably harboring gRNA targeting *NRXN2* or control gRNA and Cas13 were cultured for 0 or 2 days following the induction of differentiation. Representative images are shown. The length of the longest process was measured and depicted statistically (** $p < 0.01$; n = 100 cells). (C-F) Lysates from cells at day 0 or day 2 following the induction of differentiation were immunoblotted using antibodies against neuronal differentiation marker proteins GAP43 and β3 tubulin, or an internal control marker protein actin (left panels). The band intensity of each marker protein was normalized to that of actin and statistically analyzed (** $p < 0.01$ and * $p < 0.05$; n = 3 blots).

Figure S3. RGS3 does not significantly affect cell morphology or neuronal marker expression following the induction of differentiation. (A, B) Plasmids encoding the RGS domain of RGS3 (hereafter referred to as RGS3) or the corresponding control vector were co-transfected with Cas13 and a control gRNA plasmid into N1E-115 cells. After induction of differentiation, cells were cultured for 0 or 2 days. Representative images are shown. The length of the longest process was measured and depicted statistically (**$p < 0.01$; n = 100 cells). (C, D) Lysates from cells at day 0 or day 2 following the induction

of differentiation were immunoblotted with antibodies against neuronal differentiation marker proteins GAP43 and β3 tubulin, with actin used as a loading control. The band intensity of each marker protein was normalized to that of actin and statistically analyzed (** $p < 0.01$ and * $p < 0.05$; $n = 3$ blots). Vertical lines in the graph indicate the groups being compared.

Figure S4. GBAi, a Girdin inhibitor, does not significantly affect cell morphology or neuronal marker expression following the induction of differentiation. (A, B) Plasmids encoding GBAi or the corresponding control vector were co-transfected with Cas13 and a control gRNA plasmid into N1E-115 cells. After induction of differentiation, cells were cultured for 0 or 2 days. Representative images are shown. The length of the longest process was measured and depicted statistically (** $p < 0.01$; $n = 100$ cells). (C, D) Lysates from cells at day 0 or day 2 following the induction of differentiation were immunoblotted with antibodies against neuronal differentiation marker proteins GAP43 and β3 tubulin, with actin used as a loading control. The band intensity of each marker protein was normalized to that of actin and statistically analyzed (** $p < 0.01$ and * $p < 0.05$; $n = 3$ blots). Vertical lines in the graph indicate the groups being compared.

Figure S5. The effects of transfection of the respective G protein signaling components on primary cortical neurons. (A, B) Primary cortical neurons, which were isolated from mouse brain cortical tissues, were transfected with the plasmids encoding the respective constructs and pEGFP-C1 for morphological visualization, together with gRNA targeting

NRXN2 gRNA and Cas13, and cultured for 1 or 3 days. Representative images are shown. The length of an axonal process was measured and depicted statistically (** $p < 0.01$; n = 200 cells).

Figure S6. Amino acids 81-310 of ELMO1 (G protein-binding domain) does not significantly affect cell morphology or neuronal marker expression following the induction of differentiation. (A, B) Plasmids encoding amino acids 81-310 of ELMO1 (hereafter referred to as 81-310) or the corresponding control vector were co-transfected with Cas13 and a control gRNA plasmid into N1E-115 cells. After induction of differentiation, cells were cultured for 0 or 2 days. Representative images are shown. The length of the longest process was measured and depicted statistically (**$p < 0.01$; n = 100 cells). (C, D) Lysates from cells at day 0 or day 2 following the induction of differentiation were immunoblotted with antibodies against neuronal differentiation marker proteins GAP43 and β3 tubulin, with actin used as a loading control. The band intensity of each marker protein was normalized to that of actin and statistically analyzed (**$p < 0.01$ and *$p < 0.05$; n = 3 blots). Vertical lines in the graph indicate the groups being compared.

Figure S7. Computer-saved full-size gel images for Figure 1.

Figure S8. Computer-saved full-size gel images for Figure 2.

Figure S9. Computer-saved full-size gel images for Figure 3.

Figure S10. Computer-saved full-size gel images for Figure 4.

Figure S11. Computer-saved full-size gel images for Figure 5.

Figure S12. Computer-saved full-size gel images for Figure S1.

Figure S13. Computer-saved full-size gel images for Figure S2.

Figure S14. Computer-saved full-size gel images for Figure S3.

Figure S15. Computer-saved full-size gel images for Figure S4.

Figure S16. Computer-saved full-size gel images for Figure S6.
